# Addition of nimotuzumab to concurrent chemoradiotherapy after induction chemotherapy improves outcomes of patients with locally advanced nasopharyngeal carcinoma

**DOI:** 10.3389/fphar.2024.1366853

**Published:** 2024-03-21

**Authors:** Run-Jie Wang, Rui-Quan Ke, Yi-Feng Yu, Guan-Zhong Lu, San-Gang Wu

**Affiliations:** Department of Radiation Oncology, Xiamen Cancer Quality Control Center, Xiamen Cancer Center, Xiamen Key Laboratory of Radiation Oncology, The First Affiliated Hospital of Xiamen University, School of Medicine, Xiamen University, Xiamen, China

**Keywords:** nasopharyngeal carcinoma, anti-EGFR, nimotuzumab, outcome, radiotherapy

## Abstract

**Purpose:**

To investigate the survival outcomes and toxicities associated with the addition of nimotuzumab to concurrent chemoradiotherapy (CCRT) in locally advanced nasopharyngeal carcinoma (LANPC) patients who received induction chemotherapy (IC).

**Methods:**

Patients with stage III-IVA nasopharyngeal carcinoma who received IC and CCRT between January 2017 and October 2021 were retrospectively included. We aimed to compare the locoregional recurrence-free survival (LRFS), distant metastasis-free survival (DMFS), disease-free survival (DFS), and overall survival (OS) between patients treated with CCRT+nimotuzumab and CCRT alone.

**Results:**

We included 411 patients in the analysis. Of these patients, 267 (65.0%) and 144 (35.0%) had CCRT+nimotuzumab and CCRT alone, respectively. Similar LRFS was found between those with and without nimotuzumab (92.9% vs. 92.6%, *p* = 0.855). The 3-year DMFS was 88.2% and 76.2% in those with and without nimotuzumab (*p* = 0.002). The 3-year DFS was 83.4% and 70.6% in those with and without nimotuzumab treatment (*p* = 0.003). The 3-year OS was 92.1% and 81.1% in those with and without nimotuzumab (*p* = 0.003). The multivariate Cox regression analysis indicated that the addition of nimotuzumab was independently associated with better DMFS (hazard ratio [HR] 0.606, *p* = 0.049), DFS (HR 0.613, *p* = 0.028), and OS (HR 0.497, *p* = 0.019). No significant differences in major toxicities were found between the two treatment arms, including hematologic toxicities, hepatoxicity, nephrotoxicity, gastrointestinal reactions, and mucositis (all *p* > 0.05).

**Conclusion:**

The addition of nimotuzumab to CCRT after IC in LANPC has shown promising results in improving treatment outcomes and acceptable toxicities.

## Introduction

Nasopharyngeal carcinoma (NPC) is a prevalent form of head and neck cancer, particularly in Southeast Asia, including China (Zhang et al., 2023). Due to the insidious nature of the disease, approximately 70%–80% of patients were diagnosed with locally advanced nasopharyngeal carcinoma (LANPC) (Pan et al., 2016). Currently, the standard treatment for LANPC is induction chemotherapy (IC) followed by concurrent chemoradiotherapy (CCRT). The goal of this treatment approach is to maximize local control, reduce the risk of distant recurrence, and improve long-term survival (Chen et al., 2021). The 5-year overall survival (OS) for LANPC patients receiving IC+CCRT has reached 87% (Zhang et al., 2022). Despite advancements in treatment approaches, the management of LANPC remains challenging and outcomes vary among patients because approximately 20% of patients would develop disease recurrence (Chen et al., 2022; Tian et al., 2022). This has led researchers to explore the potential impact of targeted therapies in improving treatment outcomes.

Epidermal growth factor receptor (EGFR) signaling plays a critical role in the development and progression of several malignancies, including NPC (Xu et al., 2017; Chen et al., 2020). High expressions of EGFR are commonly found in NPC and have been associated with poor prognosis (Sun et al., 2014). Activation of the EGFR pathway has been demonstrated to promote tumor cell growth, invasion, and angiogenesis, while also inhibiting apoptosis and inducing chemoresistance and radioresistance (Sigismund et al., 2018). By binding to EGFR, the monoclonal antibody nimotuzumab inhibits the EGFR signaling pathway. The addition of nimotuzumab to the treatment regimen may have the potential to increase tumor response, reduce distant metastasis, and improve outcomes in LANPC patients (Chen et al., 2020; Liang et al., 2021). However, the existing literature showed mixed results regarding the use of nimotuzumab in LANPC (Fei et al., 2020; Jiang et al., 2023; Yang et al., 2023). The main reasons for the differences in the above results may include variations in treatment strategies and small sample sizes of the enrolled patients. In light of this, our study aimed to investigate the survival outcomes and toxicities associated with the addition of nimotuzumab to CCRT in LANPC patients who received IC.

## Materials and methods

### Patients

We conducted the present retrospective study, which included patients diagnosed with LANPC at the First Affiliated Hospital of Xiamen University from January 2017 to October 2021. Patients who met the following inclusion were included in the analysis: 1) stage III-IVA NPC based on the eighth edition of the American Joint Committee on Cancer staging system; 2) Eastern Cooperative Oncology Group performance status of 0 or 1; 3) age ≥18 years; 4) EGFR-positive disease; 5) receiving IC followed by CCRT or CCRT combined with nimotuzumab; 6) available data regarding the smoking history and alcohol history; 7) adequate haematologic, liver and renal function. Patients with a history of previous malignancy or other concurrent malignant diseases were excluded from this study. Moreover, patients who did not complete radiotherapy were also excluded. The study was approved by the Ethics Committee of the First Affiliated Hospital of Xiamen University, and informed consent was obtained from all the patients.

### Variables

The following variables were included in the analysis: age at diagnosis, gender, smoking history, alcohol history, histological subtype, clinical stage, tumor (T) stage, nodal (N) stage, IC regimen, nimotuzumab treatment as well as the plasma EBV-DNA levels before treatment. The cutoff point of Epstein-Barr virus (EBV)-DNA was 430 IU/mL, according to our previous study (Zheng et al., 2023). Former and current smokers, referred to as ever smokers, were defined as patients who had smoked within the last year or had quit smoking for more than 1 year.

### Treatment

In our institution, the IC regimens included two or three cycles of TPF (docetaxel 75 mg/m^2^ or paclitaxel 135 mg/m^2^ or nab-paclitaxel 260 mg/m^2^ on day 1, cisplatin 25 mg/m^2^ on days 1–3, and 5-FU 600–750 mg/m^2^ per day as a continuous 120 h infusion or S1 capsules 40 mg/m^2^ bid on day 1–14), TP (docetaxel 75 mg/m^2^ or paclitaxel 135 mg/m^2^ or nab-paclitaxel 260 mg/m^2^ on day 1, cisplatin 25 mg/m^2^ on days 1–3), or GP regimens (gemcitabine 1,000 mg/m^2^ on days 1 and 8, cisplatin 25 mg/m^2^ on days 1–3).

All patients in this study underwent intensity-modulated radiation therapy (IMRT). The target volumes were delineated based on the guidelines provided by the Chinese Society of Clinical Oncology (CSCO) for NPC. Specifically, the gross tumor volume (GTV), high-risk clinical target volume (CTV1), and low-risk clinical target volume (CTV2) were delineated. The total radiation dose for GTV, CTV1, and CTV2 was 70.29 grey (Gy), 62.04 Gy, and 56.10 Gy, respectively, delivered in 33 fractions given five times per week. Concurrent chemotherapy was recommended and cisplatin (80 mg/m^2^ given on days 1–3, every 3 weeks) or lobaplatin (30 mg/m^2^ on day 1, every 3 weeks) were used with two cycles. Nimotuzumab (200 mg iv, weekly for 7 courses) targeted therapy was performed in patients during CCRT. The decision-making of the induction or concurrent chemotherapy regimens was mainly according to physician-specific preference. In China, nimotuzumab has been approved for use in patients with stage III-IV NPC. In clinical practice, nimotuzumab was routinely recommended for this population during CCRT, while the decision-making of the administration of nimotuzumab mainly depended on the patient’s preference.

### Adverse reactions assessment during CCRT

The adverse reactions during CCRT were assessed according to the Common Terminology Criteria for Adverse Events (CTCAE version 5.0). We also used the toxicity criteria of the Radiation Therapy Oncology Group to record the adverse reactions regarding skin and mucosal during CCRT (Cox et al., 1995). The maximum toxicity value was recorded and evaluated.

### Follow-up

Follow-up visits were scheduled every 3 months for the first two years, followed by visits every 6 months between the 3rd and 5th year post-treatment. After that, visits were scheduled annually. During these visits, a physical examination was conducted, including an examination of the nasopharynx and neck lymph nodes. In addition, auxiliary examinations such as nasopharynx and neck MRI, endoscopy, chest CT, abdominal ultrasound, and bone emission computerized tomography were performed. PET-CT scans were also conducted when necessary. If there was suspicion of disease recurrence, a biopsy or needle biopsy was performed.

Locoregional relapse‐free survival (LRFS) was measured as the time from NPC diagnosis to the relapse of the nasopharynx or neck lymph nodes or the last follow‐up. Distant metastasis‐free survival (DMFS) was defined as the time of NPC diagnosis to the time of distant metastasis or the last follow‐up. Disease-free survival (DFS) was referred to as the time from NPC diagnosis to the date of death or first locoregional or distant recurrence. Overall survival (OS) was defined as the time of NPC diagnosis until death from any cause.

### Statistical analysis

The differences in patient characteristics between those with or without nimotuzumab were compared by the chi-square test. Survival rates were estimated using the Kaplan-Meier method, and the Log-rank test was used to compare the differences. Multivariate Cox regression analyses were conducted to identify independent prognostic factors related to survival outcomes. All statistical analyses were performed using the SPSS statistical software package (version 26.0; IBM Corporation, Armonk, NY, USA). Statistical significance was defined as a *p*-value of less than 0.05.

## Results

### Patient baseline characteristic

We included 411 patients in the analysis (Table 1). Of these patients, 300 (73.0%) were male, 352 (85.6%) had the WHO III subtype, 294 (71.5%) had stage T3-4 disease, and 308 (75.0%) had stage N2-3 disease. In those with EBV-DNA available before treatment, 176 (46.0%) and 207 (54.0%) patients had EBV-DNA <430 IU/mL and ≥430 IU/mL, respectively.

**TABLE 1:** Patient baseline characteristics between those with or without nimotuzumab during concurrent chemoradiotherapy.

Variables	n	No nimotuzumab (%)	Nimotuzumab (%)	*p*
Age (years)				
<50	211	63 (43.8)	148 (55.4)	0.024
≥50	200	81 (56.3)	119 (44.6)	
Gender				
Male	300	107 (74.3)	193 (72.3)	0.660
Female	111	37 (25.7)	74 (27.7)	
Smoking history				
No	213	72 (50.0)	141 (52.8)	0.587
Yes	198	72 (50.0)	126 (47.2)	
Alcohol history				
No	299	100 (69.4)	199 (74.5)	0.269
Yes	112	44 (30.6)	68 (25.5)	
Histology				
WHO II	59	24 (16.7)	35 (13.1)	0.326
WHO III	352	120 (83.3)	232 (86.9)	
T stage				
T1	48	9 (6.3)	39 (14.6)	0.071
T2	69	28 (19.4)	41 (15.4)	
T3	195	69 (47.9)	126 (47.2)	
T4	99	38 (26.4)	61 (22.8)	
Nodal stage				
N0	14	5 (3.5)	9 (3.4)	0.759
N1	89	27 (18.8)	62 (23.2)	
N2	179	64 (44.4)	115 (43.1)	
N3	129	48 (33.3)	81 (30.3)	
Clinical stage				
III	198	63 (43.8)	135 (50.6)	0.187
IVA	213	81 (56.3)	132 (49.4)	
EBV-DNA level before treatment (IU/mL)				
<430	176	51 (35.4)	125 (46.8)	0.077
≥430	207	81 (56.3)	126 (47.2)	
Unknown	28	12 (8.3)	16 (6.0)	

Regarding the IC regimens, there were 303 (73.7%), 59 (14.4%), and 49 (11.9%) patients receiving TP, GP, and TPF regimens, respectively. Of the patients, 115 (28.0%) and 296 (72.0%) were treated with two and three cycles of IC, respectively. All patients received CCRT, and 267 (65.0%) patients had nimotuzumab treatment. Patients aged <50 years were more likely to receive nimotuzumab during CCRT (55.4% vs. 43.8%, *p* = 0.024). Similar distribution regarding gender (*p* = 0.660), smoking history (*p* = 0.587), alcohol history (*p* = 0.269), histological subtype (*p* = 0.326), T stage (*p* = 0.071), N stage (*p* = 0.759), and EBV-DNA status before treatment (*p* = 0.077) were found between the treatment arms.

### Survival

**FIGURE 1:**
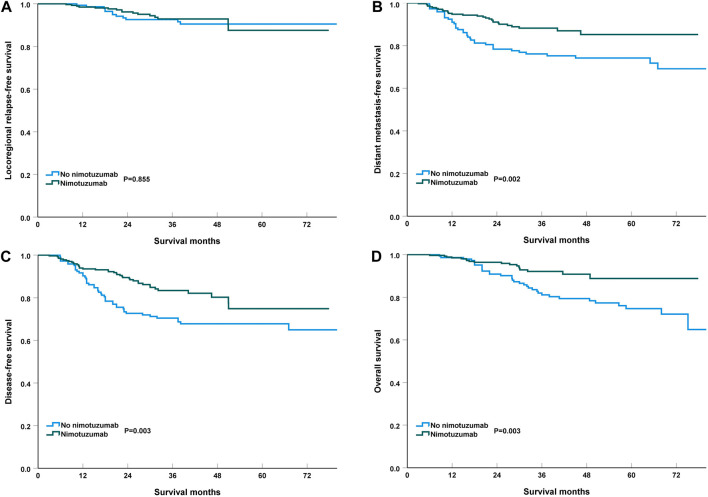
Kaplan-Meier plots of locoregional recurrence-free survival **(A)**, distant metastasis‐free survival **(B)**, disease-free survival **(C)**, and overall survival **(D)** of locally advanced nasopharyngeal carcinoma patients treated with or without nimotuzumab during concurrent chemoradiotherapy.

With a median follow-up of 35.9 months (range, 6–81 months), the 3-year LRFS, DMFS, DFS, and OS were 93.0%, 83.7%, 78.9%, and 87.3%, respectively. Similar LRFS was found between those with and without nimotuzumab during CCRT (92.9% vs. 92.6%, *p* = 0.855) (Figure 1A). Patients who received nimotuzumab during CCRT had significantly better DMFS, DFS, and OS than those treated with CCRT alone. The 3-year DMFS was 88.2% and 76.2% in those with and without nimotuzumab during CCRT (*p* = 0.002) (Figure 1B). The 3-year DFS was 83.4% and 70.6% in those with and without nimotuzumab during CCRT (*p* = 0.003) (Figure 1C). The 3-year OS was 92.1% and 81.1% in those with and without nimotuzumab during CCRT (*p* = 0.003) (Figure 1D).

### Prognostic analysis

Multivariate Cox regression models were used to determine the independent prognostic factors related to survival outcomes (Table 2). Variables included age, gender, smoking history, alcohol history, histological subtype, EBV-DNA status before treatment, T stage, N stage, and nimotuzumab treatment were entered into multivariate Cox regression models. The results indicated that the addition of nimotuzumab was independently associated with better DMFS (hazard ratio [HR] 0.606, 95% confidence interval [CI] 0.368–0.997, *p* = 0.049), DFS (HR 0.613, 95%CI 0.396–0.949, *p* = 0.028), and OS (HR 0.497, 95%CI 0.277–0.893, *p* = 0.019). However, the administration of nimotuzumab was not related to a better LRFS (HR 0.927, 95%CI 0.427–2.026, *p* = 0.849). Age, smoking history, histology, T stage, and EBV-DNA status were independent prognostic factors associated with survival outcomes.

**TABLE 2:** Multivariate Cox regression analyses of independent prognostic factors associated with survival outcomes.

Variables	LRFS			DMFS			DFS			OS		
	HR	95% CI	*p*	HR	95% CI	*p*	HR	95% CI	*p*	HR	95% CI	*p*
Age (years)												
<50	1			1			1			1		
≥50	0.687	0.313–1.509	0.350	2.298	1.362–3.878	0.002	1.496	0.964–2.321	0.072	1.484	0.829–2.656	0.184
Gender												
Male	1			1			1			1		
Female	0.937	0.335–2.625	0.902	0.600	0.329–1.094	0.096	0.687	0.370–1.274	0.233	0.680	0.266–1.738	0.421
Smoking history												
No	1			1			1			1		
Yes	0.610	0.238–1.559	0.302	1.293	0.687–2.436	0.426	1.023	0.593–1.765	0.936	2.841	1.555–5.187	<0.001
Alcohol history												
No	1			1			1			1		
Yes	1.893	0.783–4.574	0.156	0.829	0.453–1.514	0.541	1.053	0.628–1.765	0.844	0.596	0.312–1.136	0.116
Histology												
WHO II	1			1			1			1		
WHO III	0.465	0.191–1.133	0.092	0.654	0.343–1.250	0.199	0.54	0.313–0.932	0.027	0.611	0.306–1.221	0.163
T stage												
T1	1			1			1			1		
T2	0.711	0.112–4.498	0.717	5.889	1.339–25.893	0.019	2.689	0.887–8.157	0.081	5.134	0.645–40.855	0.012
T3	1.475	0.311–6.988	0.625	4.838	1.128–20.756	0.034	2.711	0.935–7.860	0.066	5.598	0.746–42.024	0.094
T4	1.164	0.205–6.606	0.864	5.746	1.253–26.350	0.024	3.261	1.051–10.124	0.041	12.06	1.561–93.158	0.017
Nodal stage												
N0-1	1			1			1			1		
N2	0.765	0.284–2.064	0.597	0.891	0.422–1.883	0.763	0.709	0.389–1.291	0.262	0.930	0.425–2.034	0.855
N3	0.906	0.303–2.711	0.86	2.138	0.994–4.601	0.052	1.339	0.743–2.413	0.331	1.955	0.840–4.549	0.120
EBV-DNA level before treatment (IU/mL)												
<430	1			1			1			1		
≥430	1.964	0.788–4.896	0.147	2.656	1.357–5.199	0.004	2.612	1.519–4.490	<0.001	2.419	1.157–5.059	0.019
Nimotuzumab												
No	1			1			1			1		
Yes	0.927	0.424–2.026	0.849	0.606	0.368–0.997	0.049	0.613	0.396–0.949	0.028	0.497	0.277–0.893	0.019

### Subgroup analysis

**FIGURE 2:**
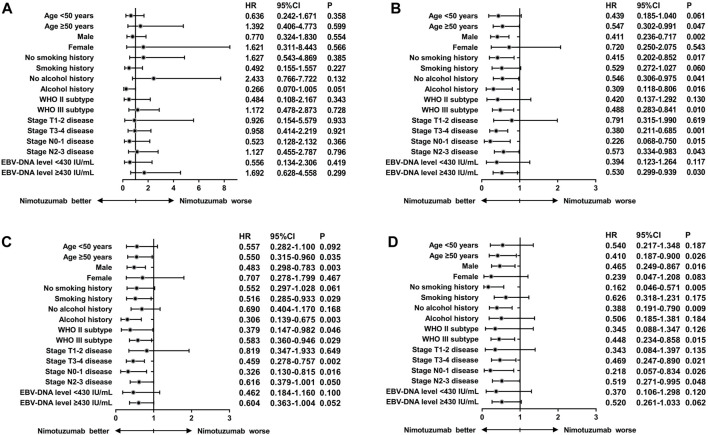
Hazard ratios for survival outcomes of locally advanced nasopharyngeal carcinoma patients treated with or without nimotuzumab during concurrent chemoradiotherapy after stratification by clinicopathological characteristics (**(A)**, locoregional recurrence-free survival; **(B)**, distant metastasis‐free survival; **(C)**, disease-free survival; **(D)**, and overall survival).

We performed subgroup analyses to investigate whether the addition of nimotuzumab to CCRT was associated with better survival outcomes in each subgroup (Figure 2). We found that those with aged ≥50 years, male, no smoking history, and no alcohol history appeared to gain more survival benefits by using nimotuzumab. In addition, patients with stage T3-4 disease and EBV-DNA level ≥430 IU/mL before treatment also gained more benefits from the addition of nimotuzumab.

### Toxicities during CCRT with or without nimotuzumab

Table 3 shows the incidence of grade 3–4 acute toxicities in patients during CCRT without or without nimotuzumab. Hematological toxicity and mucositis were the most frequently observed acute toxicities in both treatment arms. However, no significant differences in major toxicities were found between the two treatment arms, including hematologic toxicities, hepatoxicity, nephrotoxicity, gastrointestinal reactions, and mucositis (all *p* > 0.05). Overall, treatment toxicity was well-tolerated, and there were no treatment-related deaths occurred in either treatment group.

**TABLE 3:** Grade 3/4 acute toxicities during concurrent chemoradiotherapy with or without nimotuzumab.

Toxicities	No nimotuzumab		Nimotuzumab		
	Grade 3	Grade 4	Grade 3	Grade 4	*p*
Hematologic					
Leukopenia	33 (22.9)	8 (5.6)	60 (22.5)	22 (8.2)	0.607
Neutropenia	32 (22.2)	2 (1.4)	48 (18.0)	3 (1.1)	0.560
Anemia	6 (4.2)	0	15 (5.6)	0	0.524
Thrombocytopenia	2 (1.4)	0	8 (6.0)	0	0.313
Nonhematologic					
Hepatotoxicity	7 (4.9)	0	10 (3.7)	0	0.588
Nephrotoxicity	2 (1.4)	0	3 (1.1)	0	0.815
Skin reaction	9 (6.3)	1 (0.7)	19 (7.1)	2 (0.7)	0.944
Mucositis	36 (25.0)	4 (2.8)	71 (26.6)	10 (3.7)	0.805
Nausea	13 (9.1)	3 (2.1)	18 (6.7)	8 (3.0)	0.619
Vomiting	18 (12.5)	3 (2.1)	30 (11.2)	3 (1.1)	0.680

## Discussion

In this study, we explored whether the addition of nimotuzumab to CCRT would lead to improved survival outcomes of LANPC patients after IC. Our results showed that the addition of nimotuzumab was significantly associated with better DMFS, DFS, and OS of patients, but it did not improve LRFS. In addition, our study also found that the administration of nimotuzumab during CCRT was not associated with an increase in toxicities than those treated with CCRT alone.

The expression rate of EGFR in NPC patients exceeds 90%, and patients with high expression of EGFR were more likely to experience locoregional recurrence and distant metastasis (Sun et al., 2014). Activation of the EGFR pathway has been demonstrated to enhance the growth, invasion, and angiogenesis of tumor cells. It also inhibits apoptosis and contributes to the development of chemoresistance and radioresistance in cancer cells (Sigismund et al., 2018). Currently, there have been several single-arm studies attempting to explore the impact of the addition of nimotuzumab during CCRT for LANPC (Zhai et al., 2015; Zhang et al., 2018a; Zhang et al., 2018b). Due to the lack of a control group in the above studies, it is impossible to determine nimotuzumab’s efficacy in this population. In addition, several retrospective analyses have explored the efficacy of CCRT + nimotuzumab vs. CCRT alone in LANPC, and the results showed that the addition of nimotuzumab to CCRT was associated with better DMFS, DFS, and OS, but has no effect on LRFS (You et al., 2017; Yao et al., 2018; Zhi-Qiang et al., 2019; Cai et al., 2023; Lu et al., 2023). However, patients included in the above study did not undergo IC or had a lower percentage of IC receipt. In years of 2022, a prospective study reported the results at the American Society of Clinical Oncology meeting and showed that the addition of nimotuzumab to CCRT was associated with a better 5-year OS (76.9% vs. 64.3%, *p* = 0.042), but was not associated with a better 5-year LRFS (93.0% vs. 94.6%, *p* = 0.804), DMFS (81.5% vs. 84.8%, *p* = 0.500) as well as DFS (40.0% vs. 14.4%, *p* = 0.192) compared to those treated with CCRT alone (Sun et al., 2022). However, in the above study, the patients were enrolled between October 2009 and March 2012, and the patients did not receive IC. In the current clinical practice, IC+CCRT has become the standard treatment regimen for LANPC. Therefore, the treatment model of the above studies is inconsistent with current clinical practice. It is needed to determine whether increasing targeted therapy under standard treatment modes can further improve survival.

In the CSCO guidelines, nimotuzumab has been recommended for the treatment of LANPC (Tang et al., 2021). In China, nimotuzumab treatment can be reimbursed by medical insurance for LANPC patients with EGFR-positive disease. In this study, a total of 267 patients received nimotuzumab treatment, and the proportion of our patients receiving nimotuzumab was higher than in other studies (65% vs. 6.4%–38.8%) (Zhai et al., 2015; You et al., 2017; Zhang et al., 2018a; Zhang et al., 2018b; Yao et al., 2018; Zhi-Qiang et al., 2019; Fei et al., 2020; Cai et al., 2023; Jiang et al., 2023; Lu et al., 2023; Yang et al., 2023). Our study found that the addition of nimotuzumab to CCRT significantly improved DMFS, DFS, and OS in patients after two to three cycles of IC. There is currently a lack of prospective studies on the addition of nimotuzumab during CCRT in LANPC patients after IC. Several retrospective studies have explored the efficacy of the addition of nimotuzumab during CCRT after IC, but inconsistent results have been found. Yang et al. (2023) found that the addition of nimotuzumab during CCRT after IC (*n* = 52) could improve DMFS compared to those treated with CCRT alone (*n* = 57) (91.6% vs. 77.3% *p* = 0.047), but there was no significant effect on LRFS (*p* = 0.566), DFS (*p* = 0.110), and OS (*p* = 0.295). In addition, the results of multivariate analysis did not show that using nimotuzumab could improve DMFS of patients. The study by Jiang et al. (2023) did not find a better DFS and OS using multivariate analysis between those treated with IC+CCRT (*n* = 63) vs. IC+CCRT+nimotuzumab (*n* = 54). The study from Fei et al. (2020) found that the addition of nimotuzumab during CCRT after IC (*n* = 50) was associated with a better OS (*p* = 0.012) but not DFS (*p* = 0.0956) than those treated with IC+CCRT alone (*n* = 344). The number of enrolled patients in various studies is relatively small, and the adjuvant treatment mode varies, which may affect the evaluation of results. Our findings were based on the current standard treatment model for LANPC. Our study suggests that the addition of nimotuzumab to CCRT after IC could maximize survival in LANPC patients than patients taking IC+CCRT alone.

In this study, we found that distant metastasis was the main failure mode of LANPC, which was similar to the results of several previous studies based on the IMRT era (Mao et al., 2016; Chen et al., 2022; Tian et al., 2022). Our study indicated that the addition of nimotuzumab significantly improves DMFS, DFS, and OS but did not show any significant improvement in LRFS. The improvement of DMFS indicates that nimotuzumab may effectively reduce the risk of distant metastasis, which is a leading cause of treatment failure and poor prognosis in LANPC. By comprehensively investigating the effect of nimotuzumab in the context of CCRT, this study intends to provide valuable insights into the potential benefits of this treatment combination. Ultimately, the findings may contribute to optimizing therapeutic strategies and improving outcomes for patients with LANPC.

Previous studies have reported control rates of 90% for NPC when using IMRT in combination with systematic chemotherapy, even in patients with LANPC (Mao et al., 2016; Chen et al., 2022; Tian et al., 2022). In our study, we observed no significant difference in 5-year LRFS between the two treatment groups (92.9% vs. 92.6%, *p* = 0.855), which was consistent with the fact that IMRT provides excellent locoregional control and CCRT has been shown to improve locoregional control. However, the lack of significant improvement in LRFS prompts further investigation into the underlying mechanisms and potential strategies to address locoregional recurrence. There is a higher risk of local and distant recurrence for patients with stable disease and progressive disease after IC, with a 3-year locoregional recurrence rate of 19.1% and a distant recurrence rate of 24.3%, respectively (Liu et al., 2019). A study by Niu et al. (2024) showed that the addition of nimotuzumab to CCRT in patients who were resistant to IC achieved 3-year LRFS, DMFS, DFS, and OS of 80.3%, 92.9%, 79.3%, and 72.1%, respectively. These results support the rationale for future prospective studies to establish the clinical efficacy of nimotuzumab and its potential as a potential treatment approach for patients who are not sensitive to IC.

Oral mucositis is the main issue faced by anti-EGFR treatment (Xu et al., 2015; Nishii et al., 2020). The study comparing CCRT versus CCRT+ cetuximab found that adding cetuximab significantly increased the incidence of grade 3–4 oral mucositis in head and neck cancer patients (43.2% vs. 33.3%) (Ang et al., 2014). In the current study, comparable grade 3–4 mucositis was found between patients receiving CCRT plus nimotuzumab (30.3%) and patients receiving CCRT alone (27.8%). This suggests that nimotuzumab can be safely combined with CCRT for the treatment of LANPC. Several studies have also found similar results, supporting the safety of combining nimotuzumab with CCRT in LANPC (Yao et al., 2018; Zhi-Qiang et al., 2019; Fei et al., 2020; Cai et al., 2023; Yang et al., 2023). The results from the prospective study also found a similar rate of oral mucositis between those treated with CCRT+nimotuzumab and CCRT alone (*p* = 0.207) (Sun et al., 2022). Therefore, the minimization of treatment-related toxicities by using nimotuzumab may be due to its relative selectivity for tumor cells over normal cells. A study by Wang et al. (2023) found that the addition of nimotuzumab during CCRT had a significantly higher rate of hematological toxicity and acute oral mucositis compared to those treated with CCRT alone. However, the intensive concurrent chemotherapy included in the above study (taxane plus cisplatin or fluorouracil plus cisplatin) may have increased the risk of grade 3–4 mucositis during IMRT. Although concurrent nimotuzumab may not increase adverse reactions in patients who received CCRT, significant adverse reactions are still observed during CCRT. Therefore, it is important to continue pursuing low-toxicity and highly effective treatment options for LANPC. An ongoing prospective study from Yuan et al. (2022) has explored the use of nimotuzumab as an alternative to concurrent chemotherapy during radiotherapy in patients who achieve complete response or greater than 50% partial response after IC.

Several limitations should be acknowledged. First, our study was a retrospective study with inherent biases and limitations. Second, the study focused on a specific treatment regimen, and the generalizability of the results to other treatment approaches may be limited. Third, the follow-up time was relatively short, and a longer follow-up is needed to confirm the impact of nimotuzumab during CCRT on the survival of LANPC patients.

## Conclusion

In conclusion, our study suggests that the addition of nimotuzumab to CCRT after IC in LANPC has shown promising results in improving treatment outcomes and acceptable toxicities. The targeted mechanism of action with nimotuzumab enhances the efficacy of standard treatment making it a valuable therapeutic option for patients with LANPC.

## Data Availability

The raw data supporting the conclusion of this article will be made available by the authors, without undue reservation.
